# The effects of static stretching programs on muscle strength and muscle architecture of the medial gastrocnemius

**DOI:** 10.1371/journal.pone.0235679

**Published:** 2020-07-09

**Authors:** Shigeru Sato, Kakeru Hiraizumi, Ryosuke Kiyono, Taizan Fukaya, Satoru Nishishita, João Pedro Nunes, Masatoshi Nakamura

**Affiliations:** 1 Institute for Human Movement and Medical Sciences, Niigata University of Health and Welfare, Niigata City, Japan; 2 Department of Physical Therapy, Niigata University of Health and Welfare, Niigata City, Japan; 3 Department of Rehabilitation, Kyoto Kujo Hospital, Minami-ku, Japan; 4 Graduate School of Medicine, Kyoto University, Sakyo-ku, Kyoto, Japan; 5 Institute of Rehabilitation Science, Tokuyukai Medical Corporation, Toyonaka, Japan; 6 Kansai Rehabilitation Hospital, Tokuyukai Medical Corporation, Toyonaka, Japan; 7 Metabolism, Nutrition and Exercise Laboratory, Physical Education and Sport Center, Londrina State University, Londrina, Brazil; University of L’Aquila, ITALY

## Abstract

**Introduction:**

Static stretching (SS) program are widely used in clinical and athletic settings. Many previous studies investigate the effect of SS program on muscle strength and muscle architecture (muscle thickness, and pennation angleh). However, no consensus has been reached about the effect of SS programs on muscle strength and muscle architecture. The aim of this study was to investigate the effects of 6-week SS programs performed at different weekly frequencies on muscle strength, muscle thickness and pennation angle at different ankle joint positions.

**Methods:**

A total of 24 healthy male volunteers were performed 6-week SS programs (2,160 s of SS: 360 s/week*6 weeks) and were randomized to a group that performed SS once a week, or a group that performed SS three times per week. Total time under stretching was equated between groups. The muscle strength (maximum voluntary isometric contraction) at three different ankle joints were assessed before and after the 6-week SS program. In addition, muscle thickness and pennation angle were assessed by ultrasonography before and after 6-week SS program.

**Results:**

There were no significant changes in all variables before and after the 6-week SS program, regardless of weekly frequency (p > 0.05).

**Conclusions:**

Our results suggest that 6-week SS programs do not increase muscle strength or muscle architecture at different ankle joint positions, regardless of stretching frequency; however, no negative effect on these outcomes was observed, contrary to evidence on the immediate, detrimental effects of SS.

## Introduction

Static stretching (SS) is widely used in clinical setting and has been reported to increase range of motion (ROM) [1, 2] and decrease muscle stiffness of the hamstring or gastrocnemius muscles [3–5]. In athletic settings, SS program is usually performed to prevent sport injury.

Many previous studies investigating SS have reported only the acute effects on ROM, muscle stiffness, and muscle strength. Although ROM is increased immediately following SS [6, 7], muscle strength is decreased [6, 8–10]; this phenomenon is called the stretching-induced force deficit. Consistently, data from reviews demonstrate a detrimental effect of SS on muscle strength and athletic performance [11–13]; therefore, Simic *et al*. recommended avoiding the use of SS alone during warm-up routines [12]. By contrast, the effect of SS programs of at least several weeks duration on muscle strength and athletic performance remains equivocal. Interestingly, there have been no studies reporting the stretching-induced force deficit after several weeks SS program. In addition, although some studies reported that there were significant changes in muscle strength and athletic performance after SS program [14, 15], other studies reported no significant effects [16–18], which have not reached the consensus. In addition, a systematic review about the effect of stretching program on muscle strength and athletic performance concluded that more studies were needed to confirm whether stretching programs could positively affect muscle strength and performance [19].

In animal models, previous studies showed that passive stretching might trigger mechanisms that are important for muscle hypertrophy, such as insulin-like and myogenic growth factors, stretch-activated channels, the AKT/mTOR pathway and protein synthesis [20–22]. Indeed, the studies reporting chronic effects of an SS program showed robust hypertrophy and perhaps hyperplasia after several weeks of SS program intervention [23, 24]. Conversely, in human studies the muscle thickness and pennation angle are usually assessed using ultrasonography. Previous studies showed that muscle size (cross-sectional area, muscle thickness and muscle volume) has a strong influence on muscle strength [25, 26]. In addition, the pennation angle of muscle fibers also influences muscle strength [27, 28]. Taken together, these elements of muscle architecture (muscle thickness and pennation angle) could strongly influence muscle strength. Some previous studies measured these indexes before and after SS program and reported that there were no significant changes in muscle thickness, pennation angle and fascicle length of medial gastrocnemius (MG) after 3–6 week SS program [5, 16–18]. However, Freitas and Mil-Homens (2015) investigated the effect of 8-week high-intensity SS program on muscle architecture and reported significant increase in the fascicle length of biceps femoris. In addition, there were reported significant increases in the muscle thickness and fascicle length of MG after six weeks of a machine-assisted SS program [29]. Since no consensus has been reached about the effect of SS program on muscle architecture as well as muscle strength, it is necessary to reconsider the effect of SS program on muscle architecture. Furthermore, if SS program could change the muscle architecture, it is expected to have a positive effect on muscle strength. In particular, it is possible that the change in fascicle length might affect the muscle force at different angles (joint position). Therefore, along with investigating the effect of SS program on the muscle architecture, it is necessary to clarify the effect of SS program on muscle strength at different joint angles.

A SS program is considered to comprise intensity, duration, and repetition of stretches; rest intervals; and intervention frequency. In previous studies, increased duration and intensity of stretching resulted in greater increases and decreases, respectively, in ROM and passive stiffness [30–32]. In addition, Marques *et al*. (2009) found that a stretching frequency of three times per week was more effective than that of one or five times per week in increasing ROM. It is possible that stretching frequency could determine the effect of SS programs on muscle strength and architecture, in addition to ROM. However, the effects of stretching frequency on muscle strength and architecture under volume-equated conditions are unclear. This information could be useful for coaches or therapists when prescribing SS programs.

Therefore, the aim of this study was to compare the effects of two 6-week SS programs with different stretching frequencies under volume-equated conditions on muscle strength and architecture at different ankle joint positions.

## Methods

### Experimental design

A quasi-randomized controlled trial was conducted, which investigated the changes in muscle strength and architecture (defined as muscle thickness and pennation angle) in two routine SS programs with different stretching frequencies as follows: 360 s of SS conducted 1 time per week (1 time/week group) versus 120 s of SS conducted 3 times per week (3 times/week group). Muscle strength, muscle thickness, and pennation angle were measured at baseline (before intervention [PRE]) and after the 6-week SS program (after intervention [POST]) in both groups. A familiarization trial of muscle strength measurement was performed >3 days before the PRE evaluation. Following the PRE evaluation, participants were randomly allocated to either of the comparison groups in a 1:1 ratio using the alternation method. To control for immediate SS effects, all outcome measurements were performed ≥24 h after the final SS session. Participants were instructed not to initiate any other SS or strength-training programs during the study period.

### Participants

A total of 24 healthy male volunteers, who were non-athletes, participated in this study (mean ± SD; age, 20.8 ± 0.9 years; height, 168.9 ± 5.0 cm; body mass, 61.3 ± 6.2 kg). All participants engaged in sports at a recreational level, but were not involved in any regular resistance or flexibility training. All participants were fully informed of the procedures and purpose of the study, and provided written informed consent. The Ethics Committee of the Niigata University of Health and Welfare, Niigata, Japan (Procedure #17677) approved the study and complied with the requirements of the Declaration of Helsinki.

### Maximum voluntary isometric contraction

Participants were seated in an isokinetic dynamometer chair at a 0° knee angle (that is, the anatomical position) with adjustable belts fixed over their trunk and pelvis (Biodex System 3.0, Biodex Medical Systems, Inc., Shirley, NY, USA). Participants were reclined (70° hip angle and 0° full extension) to prevent tension in the posterior knee. The trunk and pelvis were firmly fixed with straps, and trunk movement was restricted by holding the handle with both hands. The maximal voluntary isometric contraction (MVIC) of the MG was measured with the ankle joint at 30° plantarflexion, in the neutral position, and at 15° dorsiflexion, which were determined by the ROM through which all participants could exert force. To obtain measurements, the ankle joint of the dominant leg was securely attached to the footplate of the dynamometer using a velcro strap. A soft cloth was inserted between the velcro strap and instep to prevent movement of the ankle joint. Two MVICs were performed for 5 s at each ankle position, and the average value of both MVICs used for analyses. Strong, verbal encouragement was provided to promote participants’ maximal effort during contractions.

### Muscle thickness and pennation angle

Participants were instructed to lie on a dynamometer table in the prone position, with their hips secured in place using an adjustable lap belt. The knee of the dominant leg was maintained in full extension, and the foot of the same leg was attached to the dynamometer footplate with adjustable belts.

B-mode ultrasonography (Aplio 500, Toshiba Medical Systems, Tochigi, Japan) with a 5–14 MHz linear probe was used to assess the muscle thickness and pennation angle of the MG. The longitudinal ultrasound image of the MG at 30% of the lower-leg length, measured from the popliteal crease to the lateral malleolus near the point of the maximal cross-sectional area of the lower leg, was obtained [33, 34]. Muscle thickness, defined as the distance between the inside edges of the fascia, and pennation angle, defined as the angle of the fascicle insertion into the deep aponeurosis, were measured with the ankle joint at 30° plantarflexion, in the neutral position, and at 15° dorsiflexion, consistent with the MVIC measurements; each measurement was performed once. Muscle thickness and pennation angle were determined using image processing software (ImageJ, National Institutes of Health, Bethesda, MD, USA). Muscle thickness was determined as the mean of the distances between the deep and superficial aponeuroses measured at both ends of each image [35, 36]. In addition, pennation angle was determined as the mean of the three fascicles as the angle between fascicle and deep aponeurosis. The test-retest reliability of the ultrasound measurements was determined by coefficient variation (CV) for eight participants. The CVs for muscle thickness were 3.7 ± 1.9% in 30° plantarflexion, 3.5 ± 2.4% in neutral position and 3.5 ± 2.4% in 15° dorsiflexion. In addition, the CVs for pennation angle were 4.0 ± 3.2% in 30° plantarflexion, 3.7 ± 2.4% in neutral position and 2.2 ± 1.8% in 15° dorsiflexion.

### Static stretching programs

Participants in both groups were instructed to perform the SS program they had been assigned to for 6 weeks using a stretching board (Asahi stretching board, Asahi Corp., Gifu, Japan). Participants stood erect with one foot on the stretching board and the other on its edge and both arms against a wall in front of the body to provide balance [16, 33]. Stretching intensity was defined as the greatest tolerated dorsiflexion angle, determined during a test conducted on the stretching board. However, participants who could tolerate >35° dorsiflexion, which was the maximal angle permitted by the stretching board, were instructed to maintain the stretching intensity by moving their body mass forward. All SS programs were performed in the laboratory under direct supervision of research team. The total weekly duration of SS was 360 s in both groups. The frequency of performing SS was every 7 days and every 2–3 days in the 1-time/week and 3-times/week groups, respectively.

### Statistical analyses

IBM SPSS Statistics version 24.0 (IBM Corp., Armonk, NY, USA) was used to conduct statistical analyses. Between-group differences in anthropometric characteristics, muscle strength, muscle thickness, and pennation angle relative to PRE evaluation values were determined using unpaired *t*-tests. For muscle strength, muscle thickness, and pennation angle, a split-plot analysis of variance (ANOVA) using two factors [time (PRE versus POST evaluation) and group (1-time/week versus 3-times/week)] was used to determine the interaction and main effects. Classification of effect size (ES) was set where ηp^2^ < 0.01 was considered small, 0.02–0.1 was considered medium and over 0.1 was considered to be a large effect size based on previous studies [37, 38]. A *post-hoc* analysis was conducted, using a paired *t*-test in each group, to determine differences between PRE and POST evaluation scores. ES were calculated as differences in the mean value between PRE- and POST-evaluation divided by the pooled standard deviation (SD) [37]. In addition, an ES of 0.00–0.19 was considered as trivial, 0.20–0.49 as small, 0.50–0.79 as moderate and ≥0.80 as large [37, 39]. Statistical significance was defined as p < 0.05. Descriptive data are reported as means ± SD.

## Results

All participants completed the SS program they were assigned to in full, and there were no drop-outs. The characteristics of study participants are reported in Table 1. There were no significant differences in age, height, or body mass between the two study groups at baseline.

**Table 1:** Characteristics of participants participating in two static stretching programs.

	One-time/week group (N = 12)	Three-times/week group (N = 12)	P-value
age (years)	21.0 ± 0.9	20.5 ± 0.8	P = 0.177
height (cm)	167.7 ± 4.2	170.0 ± 5.4	P = 0.273
weight (kg)	61.8 ± 6.1	60.8 ± 6.2	P = 0.684

Data presented as mean ± standard deviation.

### Effects of static stretching programs on maximum voluntary isometric contraction

The effects of the SS program on MVIC in both groups are reported in Tables 2 and 5. There were no significant differences between groups in PRE evaluation scores, determined by the unpaired *t*-test. The split-plot ANOVA indicated no significant interaction effects for MVIC at 30° plantarflexion or in the neutral position. There was a significant interaction effect for MVIC at 15° dorsiflexion. The *post-hoc* test revealed that there were no significant differences between PRE and POST evaluation in both groups (1-time/week, p = 0.08, ES = 0.33, and 3-times/week, p = 0.149, ES = -0.37).

**Table 2:** Values for muscle strength before and after 6-week static stretching intervention program.

(Nm)		One-time/week group (N = 12)	Three-times/week group (N = 12)	Interaction effect
MVIC at 30° plantarflexion	PRE	64.8 ± 23.4	65.7 ± 23.8	F = 0.001, P = 0.975
	POST	64.0 ± 20.4	65.1 ± 29.5	ηp^2^ = 0.000
MVIC at neutral position	PRE	172.0 ± 38.5	189.3 ± 42.1	F = 0.08, P = 0.78
	POST	169.4 ± 26.3	183.7 ± 49.8	ηp^2^ = 0.004
MVIC at 15° dorsiflexion	PRE	200.2 ± 56.1	244.2 ± 52.5	F = 5.41, P = 0.03
	POST	216.6 ±42.7	222.3 ± 65.5	ηp^2^ = 0.197

PRE, before static stretching intervention program; POST, after static stretching intervention program; MVIC, maximum voluntary isometric contraction. Data presented as mean ± standard deviation.

### Effects of static stretching programs on muscle thickness and pennation angle

The effects of the SS program on muscle thickness and pennation angle in both groups are reported in Tables 3, 4 and 5, respectively. The split-plot ANOVA indicated no significant interaction effects for muscle thickness at 30° plantarflexion the neutral position or 15° dorsiflexion. In addition, there were no significant interaction effects for pennation angle at 30° plantarflexion or the neutral position or 15° dorsiflexion.

**Table 3:** Values for muscle thickness before and after 6-week static stretching intervention program.

(cm)		One-time/week group (N = 12)	Three-times/week group (N = 12)	Interaction effect
Muscle Thickness at 30° plantarflexion	PRE	1.61 ± 0.19	1.60 ± 0.22	F = 0.589, P = 0.451
	POST	1.62 ± 0.16	1.54 ± 0.26	ηp^2^ = 0.026
Muscle Thickness at neutral position	PRE	1.68 ± 0.23	1.59 ± 0.25	F = 0.030, P = 0.865
	POST	1.66 ± 0.16	1.60 ± 0.29	ηp^2^ = 0.001
Muscle Thickness at 15° dorsiflexion	PRE	1.65 ± 0.29	1.55 ± 0.28	F = 0.021, P = 0.887
	POST	1.68 ± 0.16	1.59 ± 0.25	ηp^2^ = 0.001

PRE, before static stretching intervention program; POST, after static stretching intervention program. Data presented as mean ± standard deviation.

**Table 4:** Values for pennation angle before and after 6-week static stretching intervention program.

(cm)		One-time/week group (N = 12)	Three-times/week group (N = 12)	Interaction effect
Pennation Angle at 30° plantarflexion	PRE	22.9 ± 3.8	21.8 ± 3.3	F = 0.804, P = 0.380
	POST	21.9 ± 2.8	21.7 ± 3.2	ηp^2^ = 0.035
Pennation Angle at neutral position	PRE	19.4 ± 3.5	18.7 ± 2.4	F < 0.001, P = 0.996
	POST	19.2 ± 2.6	18.5 ± 2.3	ηp^2^ < 0.001
Pennation Angle at 15° dorsiflexion	PRE	17.7 ± 2.7	17.1 ± 2.8	F = 0.064, P = 0.803
	POST	17.1 ± 2.2	16.8 ± 2.0	ηp^2^ = 0.003

PRE, before static stretching intervention program; POST, after static stretching intervention program. Data presented as mean ± standard deviation.

**Table 5:** Effect size values for maximum voluntary isometric contraction (MVIC), muscle thickness and pennation angle according to groups after 6-week static stretching intervention program.

	One-time/week group (N = 12)	Three-times/week group (N = 12)
MVIC at 30° plantarflexion	-0.04	-0.02
MVIC at neutral position	-0.08	-0.12
MVIC at 15° dorsiflexion	0.33	-0.37
Muscle Thickness at 30° plantarflexion	0.08	-0.24
Muscle Thickness at neutral position	-0.06	0.01
Muscle Thickness at 15° dorsiflexion	0.14	0.18
Pennation Angle at 30° plantarflexion	-0.29	-0.03
Pennation Angle at neutral position	-0.05	-0.07
Pennation Angle at 15° dorsiflexion	-0.03	-0.12

Data presented as mean ± standard deviation.

## Discussion

We investigated the effects of a 6-week SS program on muscle strength and architecture of the MG at different ankle joint positions, comparing two different stretching frequencies under volume-equated conditions. We found no significant effects of the SS program on these outcomes, regardless of stretching frequency. It is important to mention that according to the literature presented in a recent review on the topic [40] and our updated knowledge, this is the first study that compared effects of different frequencies of SS on muscular adaptations that equalized the weekly volume of training. Equating the volume of training when studying training frequency is necessary to determine causality as to verify the actual influence of weekly frequency of training; otherwise, the effects of volume confound the ability to draw proper inferences [41].

In previous studies of animal models, SS programs induced muscle hypertrophy [21, 23, 24]; therefore, it was hypothesized that an SS program could produce a similar effect in humans, which could lead to increased muscle strength and athletic performance. However, our results were inconsistent with this hypothesis and revealed no significant changes in muscle thickness and pennation angle, which suggested that there was no change in fascicle length, or in muscle strength at different ankle joint positions. This discrepancy between our *in vivo* study and previous *in vitro* studies might be explained by the stretching intensity and duration of our program [42]. Nonetheless, the lack of SS effects on muscle architecture (muscle thickness, pennation angle, and fascicle length) were also reported in other previous studies [5, 16, 17, 43]. One work reported that there was a positive effect for increased muscle thickness, from Simpson *et al*. (2017), albeit with the caveat that raw data showed similar changes versus control [44, 45], calling into question the practical relevance of the findings.

One point that seems to be important to induce architectural changes in muscle is SS training intensity [46]. Although we were unable to measure stretching intensity because we used a stretching board in our SS program, the stretching intensity was expected to be much lower than the required for muscle hypertrophy, such the achieved during resistance-training. The stretching program in the previous study was carried out in a leg-press loaded with 20% of MVIC, which induced muscle hypertrophy [29]. It is possible that higher stretching intensity in the previous study contributed to the discrepancy with the current study. Nonetheless, in addition to training load, total exercise volume, defined as the product of training load and repetition number, has become recognized as an important factor for muscle hypertrophy. In this study, each participant performed 2,160 s of SS (360 s/week*6 weeks); however, the total exercise volume might be insufficient to induce muscle hypertrophy because the SS intensity and duration were low. For instance, in studies from Freitas and Mil-Homens (2015) and Simpson *et al*. (2017), which verified increases in fascicle length, the total time under stretching of the SS programs were of approximately 11,250 s (450 s*3.1 ± 0.8 sessions per week*8week) or 5,400 s, respectively; too higher than performed in our study.

Chen *et al*. (2011) reported a significant increase in concentric hamstring strength following an 8-week SS program, whereas Nakao *et al*. (2019) found no significant changes in isometric and concentric hamstring strength, but a significant increase in the peak angle of concentric strength after a 4-week SS program. In addition, Freitas and Mil-Homens (2015) reported a significant increase in the fascicle length of the biceps femoris following an 8-week high-intensity SS program. These studies demonstrate that, in the case of hamstring, an SS program may change the muscle architecture (e.g., muscle thickness or pennation angle), which may result in increased strength or performance. Among studies evaluating the gastrocnemius muscle, Akagi *et al*. (2014) reported no significant changes in MVIC and muscle thickness And Blazevich *et al*. (2014) reported no significant change in fascicle length after a 3-week SS program; the latter finding was supported by Nakamura *et al*. (2012) and Konrad *et al*. (2014). Previous studies have noted the difference in muscle architecture (muscle thickness, pennation angle, fascicle length and tendon length) between the biceps femoris and medial gastrocnemius muscle [47, 48]. This could affect differences arising in prior literature on the chronic effect of SS training. Collectively, these results suggest that the effects of SS training on muscle strength and architecture may differ on the basis of the target muscle and total exercise volume (stretching intensity and duration). Therefore, future studies should elucidate the variation in effects of SS programs on strength and architecture of different muscles.

An important factor regarding changes in muscle architecture might be nutrition. Our study did not incorporate a nutritional component to the SS program. However, Simpson *et al*. (2017) reported increased MG thickness and fascicle length after a 6-week SS program which included subsequent protein intake, suggesting this might have moderated the hypertrophic effect of the SS program. Future studies should consider the combined effects of SS programs, nutritional interventions, and resistance training.

This study has several limitations. The major limitations of this study were the small sample size and the short duration of the intervention, although it is similar to several previous studies on SS [5, 16, 17, 29, 43]. In this study, we calculated the sample size needed for split-plot ANOVA (alpha error = 0.05, power = 0.80, effect size = 0.4 [large]), and the requisite number of participants was 14 in each group. Therefore, it is feasible to be underpowered in this study. We calculated the effect sizes for the split-plot ANOVA (ηp^2^) and different between PRE and POST in each group. As the results of effect sizes, all variables were trivial or small. Therefore, we have assumed that the results of this study have not been underpowered. However, further study is needed to investigate the chronic effect of SS programs on muscle strength and muscle architecture using more subjects for a longer duration. In addition, the effects of SS programs on muscle performance (for example, jumping or sprinting) should be considered. In addition, the effect of a 6-week SS program on athletes is not known, and future studies are needed to investigate the effect of long-term SS program on muscle performance in other populations, such as athletes.

## Conclusion

In conclusion, a 6-week SS program targeting the MG muscle did not increase muscle strength or hypertrophy; however, we also found no negative effect on these outcomes, contrary to evidence on the immediate, detrimental effects of SS.

## Supporting information

S1 Data (XLSX)
